# Monitoring neural activity during sleep/wake events in adult *C. elegans* by automated sleep detection and stimulation

**DOI:** 10.1016/j.xpro.2022.101532

**Published:** 2022-07-13

**Authors:** Daniel E. Lawler, Dirk R. Albrecht

**Affiliations:** 1 Department of Biomedical Engineering, Worcester Polytechnic Institute, Worcester, MA 01609, USA; 2 Department of Biology and Biotechnology, Worcester Polytechnic Institute, Worcester, MA 01609, USA

**Keywords:** Microscopy, Model Organisms, Neuroscience, Behavior

## Abstract

Sleep in adult *C. elegans* occurs spontaneously, making timing of individual sleep/wake state transitions unpredictable. This protocol presents a closed-loop system to automatically detect sleep state transitions, trigger stimulation, and record evoked neural responses. This allows users to assess functional consequences of behavioral states in an unbiased manner and identify state-dependent neuromodulation. This closed-loop system is flexible and can be configured to detect any visible events, such as behavior patterns or optical reporters, and measure corresponding evoked neural responses.

For complete details on the use and execution of this protocol, please refer to Lawler et al. (2021).

## Highlights

• Protocol to assess neural activity changes between sleep and wake states in *C. elegans*
• Closed-loop system automatically detects sleep state and triggers neural stimulation
• Microfluidic arenas enable recording of freely moving behavior and neural responses
• Image analysis software extracts sleep-dependent neural changes

## Abstract (editor highlights)

Publisher’s note: Undertaking any experimental protocol requires adherence to local institutional guidelines for laboratory safety and ethics.

## Graphical abstract

**Figure:**
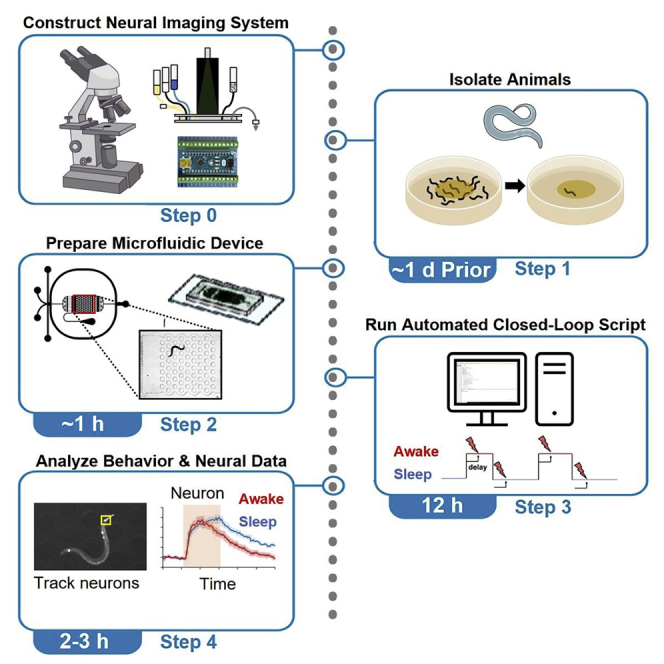

## Before you begin

### Construct the neural imaging system

**Timing: Several days to weeks prior to experimentation, depending on equipment availability**

**Figure 1:**
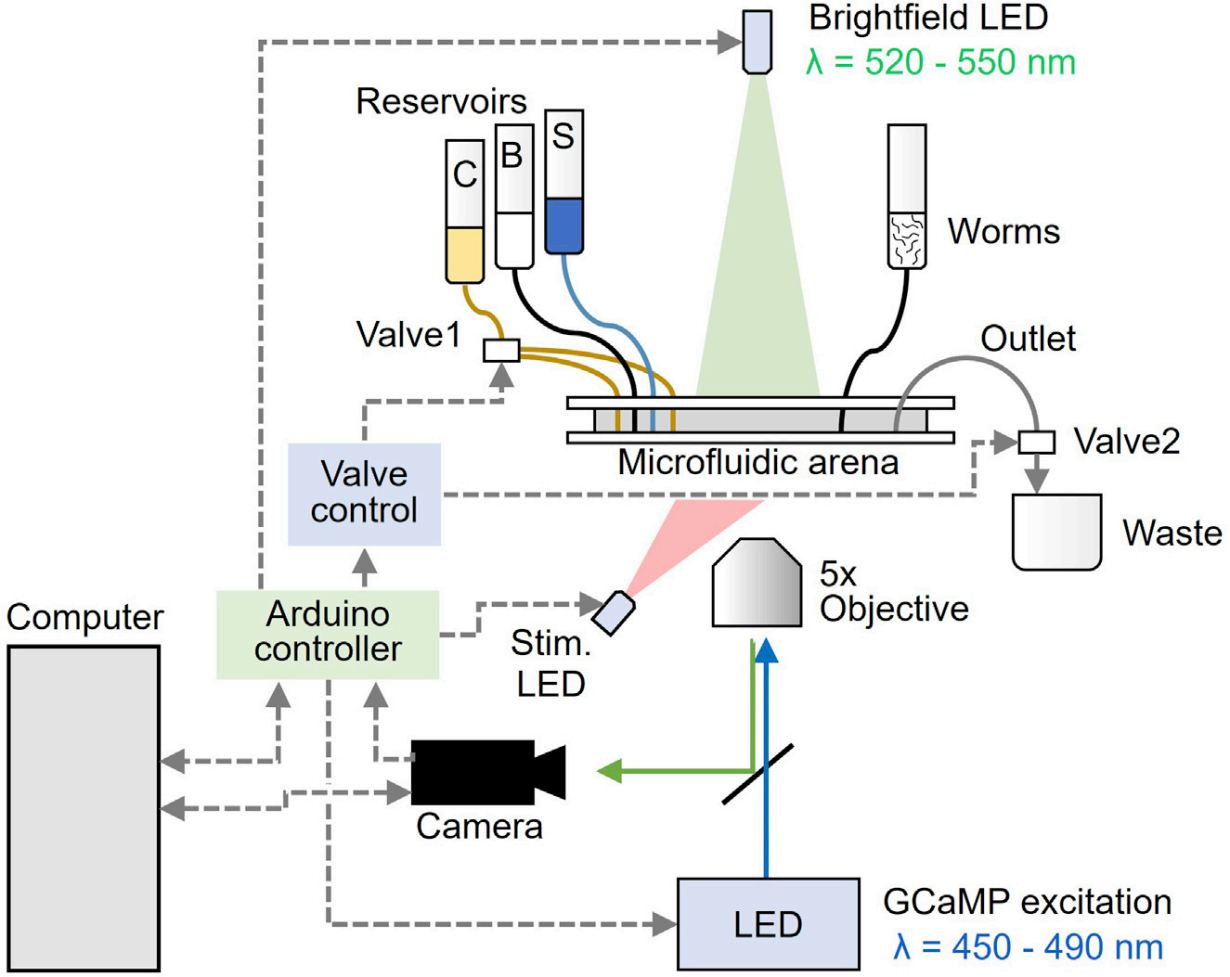
Schematic of the closed-loop neural recording setup for sleep/wake response tracking Video recording, stimulation, and LED illumination are controlled using an Arduino microcontroller. Chemical stimulation through the microfluidic arena switches between stimulus (S) and buffer (B) solutions via control fluid flow (C). Brightfield images are used to track sleep behavior and fluorescent images are used to measure GCaMP calcium transients in neurons. Image capture, sleep/wake state determination, and chemical stimulation are controlled by a computer in a closed loop, without user intervention.

A custom imaging and stimulation system controls illumination timing, image acquisition, sleep state identification, and stimulus delivery (Figure 1). Although a variety of equipment can accomplish these tasks, here we describe the construction of an inexpensive system using open-source hardware and software. An Arduino Nano microcontroller controls fluidic valves through a ValveLink 8.2 (AutoMate Scientific) valve controller and controls illumination sources for brightfield and fluorescent imaging via digital signals. The Arduino controller interacts with the camera to synchronize stimulation and image recording, as directed by open-source Micro-Manager software (Edelstein et al., 2014).

1. Build an Arduino controller with all necessary connections to the brightfield and fluorescent illumination sources, the camera, and to the valve controller (Figures 2A and 2B).
  a. Connect Arduino Nano and screw terminal breakout (Figure 2C). Solder headers onto Nano if necessary.
  b. Connect BNC screw terminals (or equivalent connectors) for the following signals, connecting the negative terminal to ground (GND) and positive to the indicated pin number (Figure 2D).
    i. CAM_IN (pin 2): Digital input signal from microscope camera indicating image frame exposure.
    ii. FL_OUT (pin 7): Digital output signal to fluorescent LED excitation.
    iii. BF_OUT (pin 8): Digital output signal to brightfield LED illumination.
      ***Optional:*** LED_OUT (pin 5): Analog output signal to optogenetic stimulation LED.
  c. Connect DB9 screw terminals for the ValveLink valve controller (Figure 2D).
    i. GND to DB9_pin1.
    ii. A0 to DB9_pin5 (valve 1) for stimulus switching.
    iii. A1 to DB9_pin9 (valve 2) for optional outflow control.
    iv. Optionally connect other valve control wires as needed, such as A2 to DB9_pin4 (valve 3). Up to eight valves can be supported to deliver multiple stimuli.
  d. Recommended: secure all cables and components.
    i. Attach Nano to ValveLink using velcro hook-and-loop fastener (Figures 2E–2G), and any others such as a LED controller.
    ii. Route all cables and secure them to ensure stable connections, e.g., using tape, zip tie, or Velcro.

2. Connect valve cable (RCA or as needed by controller) to a 3-way actuated fluidic valve for stimulus selection at valve 1.

**Figure 2:**
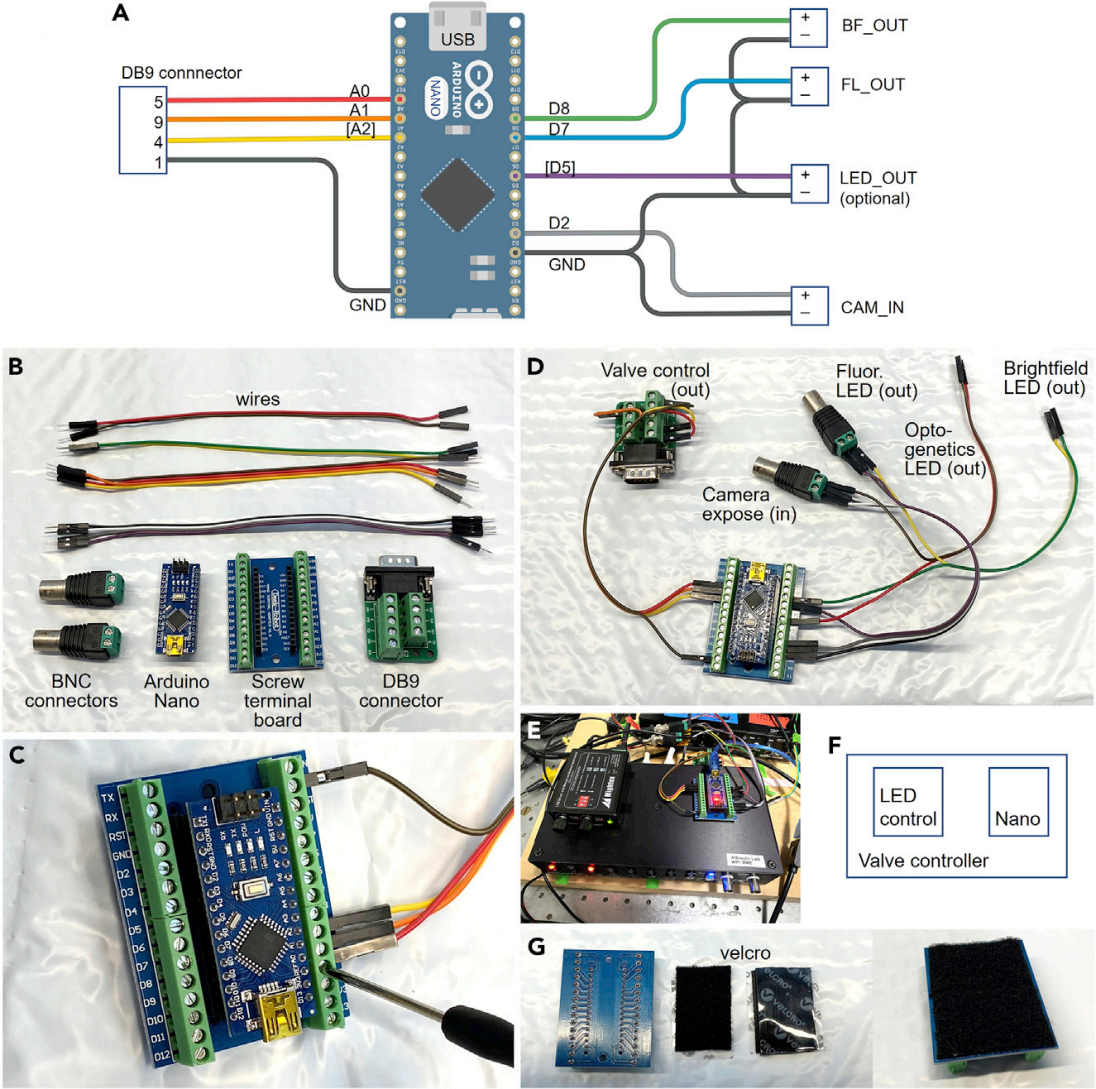
Building the Arduino controller (A) Wiring diagram of the controller. (B) Materials needed: BNC screw connectors, Arduino Nano, Screw terminal board, DB9 connector, and wires. (C) Attaching wires using the screw terminal board. (D) Completed Arduino controller showing connections to valve control, camera, and LEDs. (E) Image of Arduino controller positioned on the Valvelink valve controller. (F) Top view of Valve controller with mounted Arduino Nano and optional LED controller for optogenetic stimulation. (G) Velcro placed on the bottom of the Screw terminal board to attach securely to the Valvelink valve controller.
3. Connect valve cable (RCA or as needed by controller) to a pinch valve for outflow shutoff at valve 2.

***Alternatives:*** Other equipment can be used for each of the imaging system components, as long as any substitutes maintain the ability to control illumination timing, fluidic valves, and image acquisition. For example, the Valvelink controller can be replaced with an inexpensive multichannel relay module or MOSFET trigger switch module to actuate fluidic valves. In the configuration described, all inputs and outputs are TTL-compatible voltage signals (0–5 V). Substitution of different cameras and controllers may require modification of the open-source code, for example to invert the polarity of a control signal.

### Install and configure software

**Timing: 1–2 h for software download and installation**

Several scripts are necessary to install prior to experimentation, available at https://github.com/albrechtLab/ClosedLoopStimulation.

4. Download the “**NanoController.ino**” program and upload it to the Arduino Nano using Arduino IDE software (https://www.arduino.cc/en/software).
5. Download and install the open-source microscopy software “**Micro-Manager**” (https://micro-manager.org).
6. Configure Micro-Manager to communicate with the camera and the Arduino Nano controller.
  a. Ensure all devices are turned on, open Micro-Manager, and create a new hardware configuration. Open Tools -> Hardware Configuration Wizard… then select “Create new configuration.”
  b. Add the camera and any other microscope controllers, such as stages and shutters.
  c. Add the Arduino Nano controller using “FreeSerialPort” to add a Free-form communication Serial Port. Title the device “**ArduinoValveControl**” and select the appropriate serial port for the Arduino device (e.g., COM3 for Windows PC). Port information is accessible within the Arduino IDE (Tools -> Port) or Windows Device Manager.
  d. Save the configuration file with a recognizable name and load it at the beginning of each experiment.
7. Download the “**SleepDetection.ijm**” ImageJ Macro file. This macro analyzes bright field images and determines when a sleep state change has occurred. Copy this and other files to a folder and note the file path or location.
8. Download the “**User Defined Closed Loop Settings.txt**” text file. This text file contains experiment configuration parameters and should be adjusted as needed for each experiment.
9. Download the “**ClosedLoopSleepStim.bsh**” Micro-Manager script and add the script to the “Script Panel” within the Micro-Manager “Tools” drop-down menu. This script reads an experiment settings file and controls the Arduino and image acquisition.
10. Configure the “ClosedLoopSleepStim.bsh” script. Edit the header lines with full file locations of the “SleepDetection.ijm” macro and the “User Defined Closed Loop Settings.txt” text file.

***Note:*** This protocol was tested with MicroManager version 1.4.17. Use this version to ensure consistency of operation.

### Prepare microfluidic equipment

**Timing: 2–6 h for fabrication of new components; ∼30 min to clean and prepare reused components**

Experiments are conducted using microfluidic chambers that facilitate precise chemical stimulation and keep animals within the microscope field of view. For complete details regarding the design, fabrication, cleaning, and preparation of microfluidic devices for behavioral and neural imaging of *C. elegans* please refer to Lagoy and Albrecht (2015). For video protocols regarding the setup and use of microfluidic devices, please see Reilly et al. (2017). The reusable microfluidic device, reservoirs, and tubing should be fabricated in advance and cleaned prior to experimentation.

**Figure 3:**
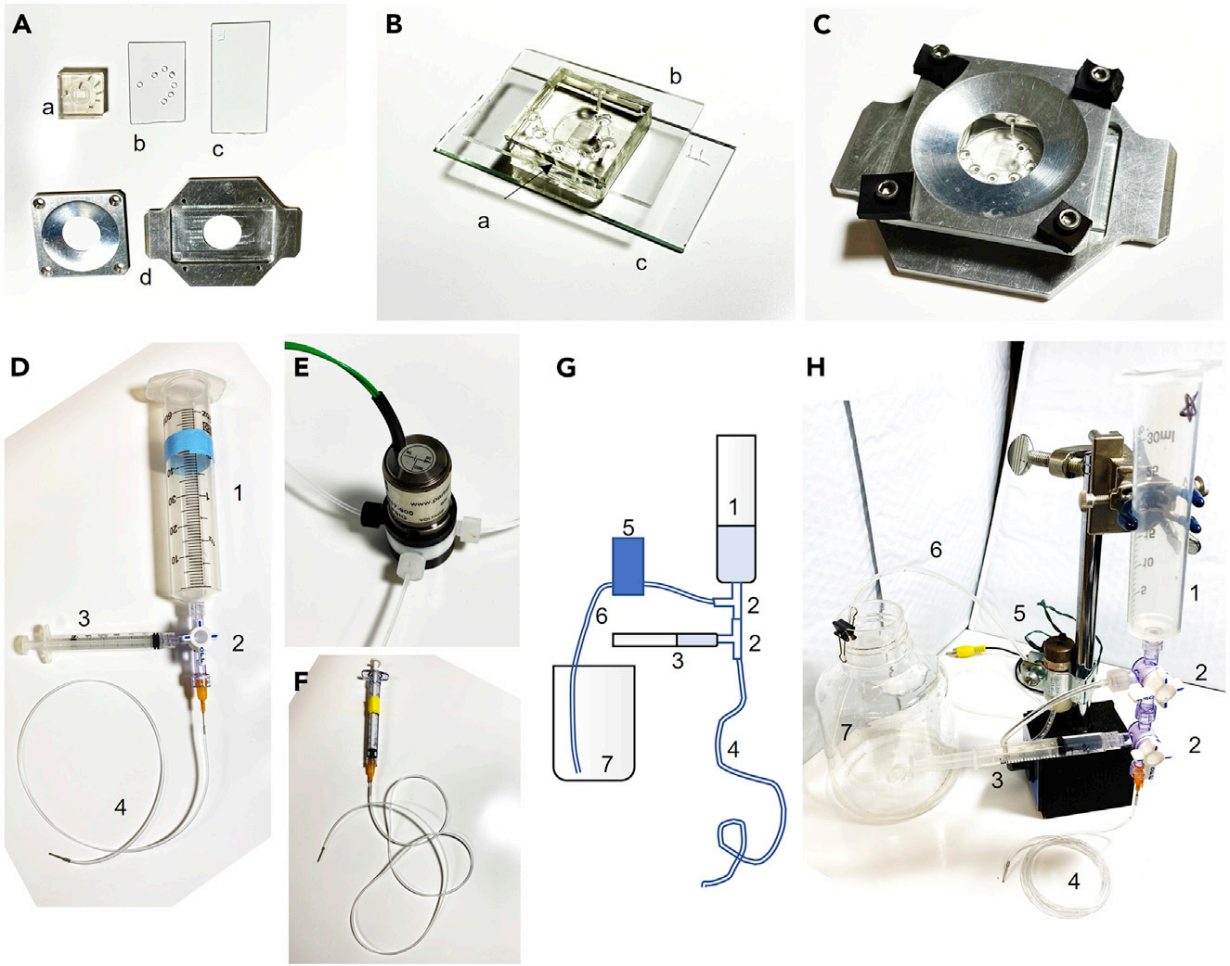
Microfluidic setup (A) Pictured are the neural imaging microfluidic device (a), the drilled top glass piece (b), the hydrophobic bottom glass piece (c), and the metal device holder (d). (B) The microfluidic device is sandwiched between top and bottom glass pieces. (C) Device secured in a clamp. (D) Microfluidic reservoir constructed from a 60 mL syringe for fluid storage (1), 3-way Luer valve for reservoir control (2), a 3 mL syringe for fluid priming (3), and tubing (4). (E) Three-way actuated fluidic valve for switching between flows during the automated neural imaging trials. (F) Worm loading syringe. (G and H) Schematic and photo of the outflow assembly consisting of a fluid reservoir (1), two Luer valves (2), a priming syringe (3), tubing to the device (4), the outflow pinch valve (5), tubing to waste (6), and a waste container (7).
  b. Drilled glass top slide (Figures 3A and 3B). Cut glass slides to size (e.g., 25 × 40 mm) with a diamond scribe and grind holes (∼2 mm diameter) using a rotary tool and diamond-coated bit.
  c. Hydrophobic (fluorinated) glass bottom slide (Figures 3A and 3C). Silanize glass with TFOCS by vapor deposition in a fume hood vacuum chamber (see Lagoy and Albrecht, 2015). Sealing hydrophobic PDMS microfluidic devices with hydrophobic glass reduces the risk of leakage.
  d. Completed microfluidic device, sandwiched between the top and bottom glass (Figure 3B) and secured in a clamp (Figure 3C).
  e. Three solution reservoirs using 60 mL syringes for fluid storage, 3 mL syringes for fluid priming, 3-way Luer valves for flow control, and tubing attached (Figure 3D). Two reservoirs (for saline buffer and copper chloride stimulus solution) have tubing attached for direct flow into the microfluidic device. One reservoir (for control solution) will connect to a 3-way electrically-actuated fluidic valve (Figure 3E) for switching between flows during the automated neural imaging trials. Label each reservoir clearly for intended use.
  f. Worm loading syringe (Figure 3F) consisting of a 1-mL syringe connected to ∼50 cm tubing and a metal end tube.
  g. Outflow tubing connected through an optional outlet pinch valve to a 10 mL or 30 mL syringe reservoir and 3 mL side syringe for priming via two 3-way Luer valves (Figures 3G and 3H). The pinch valve allows automatic flow stoppage at the end of an experiment.

***Note:*** Inlet tubing can be flexible Tygon or rigid Teflon. Consult Lagoy and Albrecht, 2015 for details. Teflon tubing is more inert and preferred for hydrophobic stimuli.

***Note:*** Components can be reused after thorough cleaning. Flush with diH_2_O, 70% ethanol, then diH_2_O, and dry using an air stream. Microfluidic devices should be soaked in 70% ethanol for 1 h to overnight and can be reused dozens of times. Devices used with hydrophobic stimuli at high concentrations may require stronger cleaning, dedicated components, or more frequent replacement.

11. Prepare all components for the microfluidic system:
  a. Neural imaging microfluidic device made from cured poly(dimethyl siloxane) (PDMS) with dermal punched inlet and outlet channel holes (Figure 3A, a). See Lagoy and Albrecht, 2015 for fabrication information, and Larsch et al. (2013) for device design.

### Prepare nematodes

**Timing: 10–20 min, timed 16–24 h prior to experiment**

Maintain *C. elegans* under standard conditions on Nematode Growth Medium (NGM) plates and fed OP50 *Escherichia coli* bacteria (Stiernagle, 2006). For neural imaging of the ASH aversive sensory neuron, use a *C. elegans* strain expressing GCaMP in the ASH neuron such as: CX10979, kyEx2865 [Psra-6::GCaMP3; Pofm-1p::GFP] (Larsch et al., 2013). Each experiment uses a single young adult *C. elegans*. Pick several array-positive L4 stage hermaphrodite animals 16–24 h prior to the experiment and isolate them to a fresh seeded plate. Adjust timing as needed for the desired stage of testing.

***Note:*** The closed-loop system is applicable to studying any neuron(s). Substitute an appropriate strain expressing GCaMP in the desired neurons. For other color indicators, such as red-emitting RCaMP, use an appropriate fluorescent excitation source and microscope filter set, and a brightfield illuminator that matches the emission filter color.

***Note:*** When using animals larger or smaller than wild-type young adult hermaphrodites, such as males, older adults, younger larval stage animals, or some genetic mutants, use microfluidic devices scaled proportionally to body size for smooth locomotion and to prevent escape from the arena.

## Key resources table

**Table:** 

REAGENT OR RESOURCE	SOURCE	IDENTIFIER
**Bacterial and virus strains**		

*E. coli* (OP50)	*Caenorhabditis* Genetics Center (CGC)	OP50

**Experimental models: Organisms/strains**		

Young adult *C. elegans* hermaphrodites expressing GCaMP in desired neurons	*Caenorhabditis* Genetics Center (CGC) or corresponding authors of published work	N/A
*C. elegans* ASH::GCaMP3 strain	Larsch et al. (2013)	CX10979

**Chemicals, peptides, and recombinant proteins**		

HEPES	Sigma-Aldrich	Cat# H3375
Magnesium chloride, MgCl_2_	Sigma-Aldrich	Cat# M2393
Calcium chloride, CaCl_2_	Sigma-Aldrich	Cat# C3881
Sodium chloride, NaCl	Sigma-Aldrich	Cat# S7653
Potassium chloride, KCl	Sigma-Aldrich	Cat# P3911
Copper chloride, CuCl_2_	Sigma-Aldrich	Cat# 751944
D-glucose	Sigma-Aldrich	Cat# G7021
Nematode Growth Medium (NGM) agar	Stiernagle (2006)	N/A
Poly(dimethyl siloxane) (PDMS)	Ellsworth Adhesives	Sylgard 184
(tridecafluoro-1,1,2,2-tetrahydrooctyl)trichlorosilane (TFOCS)	Gelest	CAS# 78560-45-9
Fluorescein, Sodium salt	Sigma-Aldrich	Cat# F6377

**Software and algorithms**		

Custom control code: ClosedLoopStimulation	Lawler et al. (2021) and this work	https://albrechtlab.github.io https://github.com/albrechtLab/ClosedLoopStimulation 10.5281/zenodo.6629830
Custom analysis code: Neurotracker	Larsch et al. (2013)	https://github.com/albrechtLab/Neurotracker 10.5281/zenodo.6632736
Micro-manager	Micro-manager	https://micro-manager.org/
MATLAB	MathWorks	https://www.mathworks.com/products/matlab.html
Arduino IDE	Arduino	https://www.arduino.cc/en/software
ImageJ	Schneider et al. (2012)	https://imagej.nih.gov/ij/

**Other**		

ValveLink 8.2 digital/manual valve controller	AutoMate Scientific	Cat #01-18
Arduino nano	Arduino	Cat #A000005
Wires and connectors	Various	See Figure 2B
3-way miniature inert PTFE isolation valve	Parker	Cat #001-0017-900
Pinch valve, normally open	NResearch	Cat #161P021
SOLA light engine (fluorescence)	Lumencor	Cat #SM6-LCR-SA
Axio Observer.A1 inverted microscope set up for epifluorescence (GFP filter cubes, 5× objective or similar)	Zeiss	Cat #491237-0012-000
Illuminator microLED (brightfield)	Zeiss	Cat #423053-9072
Orca Flash 4.0 Digital sCMOS camera	Hamamatsu	Cat #C11440-22CU
Optogenetic stimulation LED and controller (optional)	Mightex	Cat #PLS-0625-030-S and #SLA-1200-2
Petri dishes (60 mm)	Tritech	Cat #T3305
Glass slide, 1 mm thick	VWR	Cat #75799-268
Glass scribe	Ted Pella	Cat #54468
Diamond drill bit	Dremel	Cat #7134
Microfluidic device	Larsch et al. (2013); Lagoy and Albrecht (2015)	N/A
Microfluidic tubing, 0.02″ ID	Cole-Parmer	Cat #EW-06419-01
Luer 3-way stopcock	Cole-Parmer	Cat #EW-30600-07
Luer 23 ga. blunt needle	VWR	Cat #89134-100
Tube 19 ga., 0.5″	New England Small Tube	Cat #NE-1027-12
Microfluidic device clamp	Warner Instruments (or machine shop)	P-2

## Materials and equipment

**Table:** Saline buffer

Reagent	Final concentration	Amount
NaCl	80 mM	4.68 g
KCl	5 mM	0.373 g
D-glucose	20 mM	3.60 g
HEPES	10 mM	2.38 g
MgCl_2_	5 mM	1.02 g
CaCl_2_	1 mM	0.111 g
ddH_2_O	n/a	to 1 L
pH to 7.2	n/a	n/a
**Total**	**n/a**	**1 L**

Store at room temperature (15°C–25°C) for up to 1 year.

**Table:** Control buffer

Reagent	Final concentration	Amount
Saline buffer	n/a	60 mL
Fluorescein	1 μg/mL	n/a
**Total**	**n/a**	**60 mL**

Prepare fresh on day of experiment and keep at room temperature (15°C–25°C).

**Table:** Copper chloride solution

Reagent	Final concentration	Amount
Saline buffer	n/a	60 mL
1 M CuCl_2_	1 mM	60 μL
**Total**	**n/a**	**60 mL**

Prepare fresh on day of experiment and keep at room temperature (15°C–25°C).

**Table:** Nematode Growth Medium Agar

Reagent	Final concentration	Amount
NaCl	50 mM	3 g
Agar	50 mM	17 g
Peptone	10 mM	2.5 g
5 mg/mL cholesterol in ethanol	∼13 mM	1 mL
1 M KPO_4_ buffer pH 6.0	25 mM	25 mL
1 M MgSO_4_	1 mM	1 mL
1 M CaCl_2_	1 mM	1 mL
ddH_2_O	n/a	975 mL
**Total**	**n/a**	**∼1 L**

Mix dry ingredients and water and autoclave on liquid cycle. When cooled to 55°C, add the four liquid additives, mix, and pour into Petri dish plates under sterile conditions. Store plates at room temperature (15°C–25°C) for several weeks or at 4°C for several months.

## Step-by-step method details

### Prepare microfluidic system for experiment

**Timing: ∼1 h**

This step prepares the microfluidic equipment and solutions for experimentation. For complete details regarding the design, fabrication, cleaning, and preparation of microfluidic devices for behavioral and neural imaging of *C. elegans* please refer to Lagoy and Albrecht (2015). For video protocols of microfluidic device setup and operation, see Reilly et al. (2017). This example uses Copper chloride solution to stimulate the ASH aversive neurons.

1. Clean microfluidic device and top and bottom glass pieces by rinsing sequentially with water and 70% ethanol, wiping with a lint-free wipe, then drying in an air stream. Remove any dust with transparent tape, then align and secure with binder clips or a microscope slide holder (Figures 3B and 3C).
2. Place device in a vacuum desiccator for 30 min.
3. Prepare 200 mL of Saline buffer and use it to prepare 60 mL of Control buffer (1 μg/mL fluorescein) and 60 mL of Copper chloride stimulus solution (1 mM CuCl_2_).
4. Attach 3 reservoirs to the reservoir rack stand above the microscope (Figure 4A).

**Figure 4:**
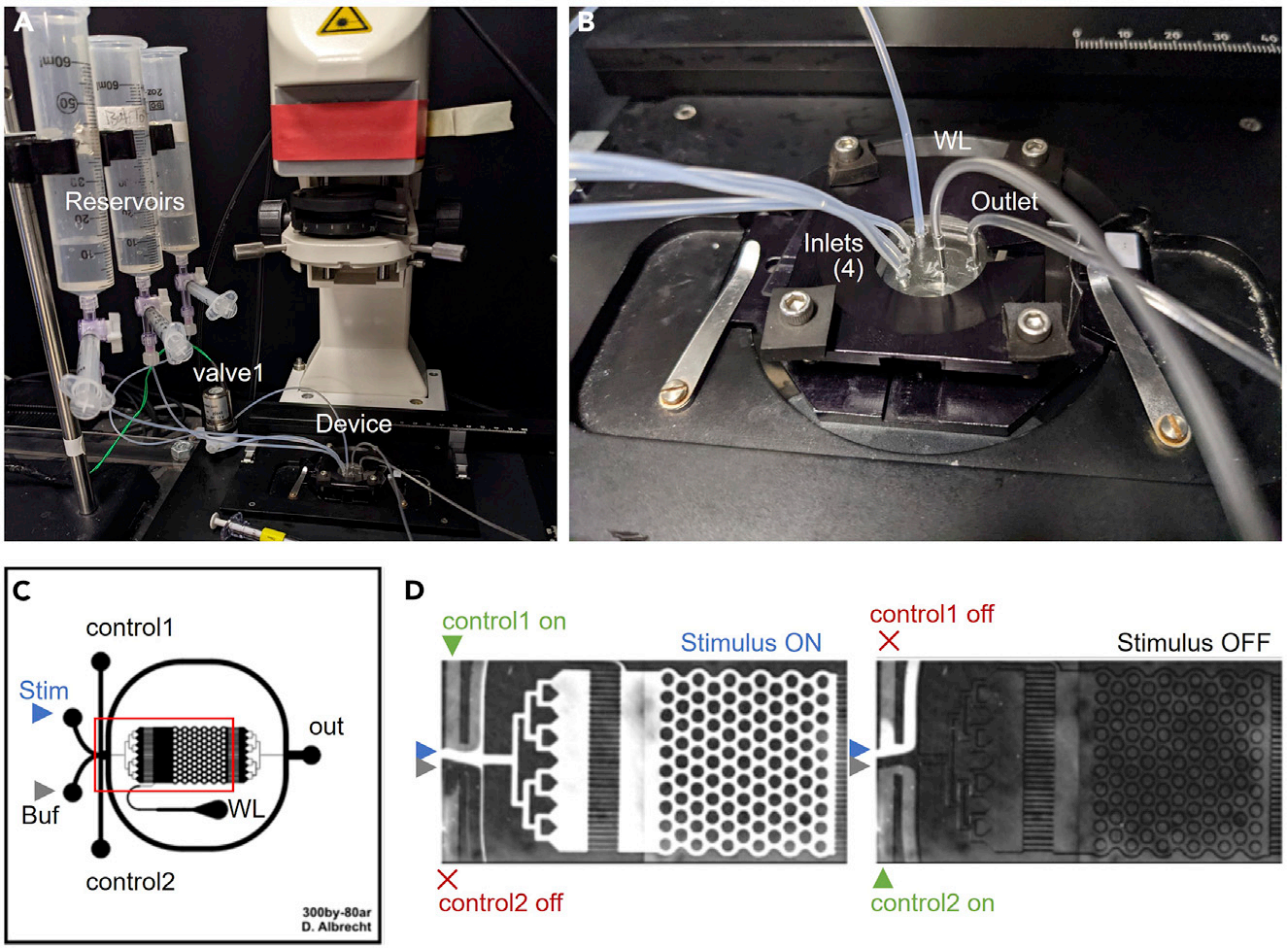
Microfluidics and microscope imaging set up (A) Reservoirs are held over the device with a tubing rack stand. Tubing from the reservoirs is connected either directly into the device or through the 3-way actuated valve for fluidic control. The device is positioned above the objective on the microscope. (B) Close up image of the microfluidic device with all tubing attached in the inlets, worm loading (WL) port, and the outlet. (C) Schematic of the 20 x 20 mm microfluidic device. Black lines represent 70 μm tall fluid channels. Stimulus (Stim) and Buffer (Buf) streams flow continuously, whereas only one control fluid inlet flows at any time. (D) Actuating the 3-way valve directs control fluid to the control1 inlet and the stimulus into the arena (left). Otherwise, control fluid enters at the control2 inlet and buffer enters the arena (right). Proper flow balance is shown, with fluorescein dye used as the stimulus fluid. Note that some of the fluid entering the arena also bypasses it via the upper and lower curved channels. These streams should be narrow and have equal width in the upper and lower channels. Adjust reservoir heights as needed.
5. Fill each of the 3 reservoirs with their designated solutions (60 mL of Saline buffer, Control buffer, or Copper chloride solution) and purge all bubbles from the connected tubing using the priming syringe (see Reilly et al., 2017 and troubleshooting Note 1). Actuate the valve to fill all tubes connected to the 3-way fluidic valve.
6. Fill the waste reservoir with ≥10 mL Saline buffer and use the priming syringe to fill both the outflow waste tube and the outflow outlet tube with buffer (see Figures 3G and 3H).
7. After 30 min in the vacuum desiccator, remove the device and mount it on the microscope.
8. Fill microfluidic device with fluid.
  a. Insert the outflow tubing into the waste outlet of the microfluidic device.
  b. Ensure the outflow valve is open and gently push buffer through the device using the priming syringe.
  c. When liquid droplets form at all 4 of the inlets, close the outflow valve (Figures 4B and 4C).
9. Insert tubing or a blocking pin into the worm loading inlet.
10. Gently insert each reservoir’s tubing into the appropriate inlet (Figures 1 and 4C).
  a. Connect the control reservoir 3-way valve (Figure 3E) “normally-closed” outlet to the “control1” inlet and its “normally-open” outlet to the “control2” inlet.
  b. Insert tubing approximately 3 mm into the PDMS inlet hole (to ∼2/3 depth).
  c. Use “drop-to-drop” connections, in which a droplet hangs from the tubing end and is connected to a liquid droplet above the microfluidic port (see Lagoy and Albrecht, 2015).
  d. Remove any excess liquid from the glass by vacuum aspiration or paper towel.
11. Open the outflow valve to establish flow. View live images in the microscope and verify that no bubbles are present in the device channels (see troubleshooting Note 1).
12. Balance inlet flows.
  a. Focus on the intersection of inlet channels (Figures 4C and 4D) and observe flow patterns while alternately activating and deactivating the 3-way stimulus valve.
  b. Adjust reservoir heights up or down so that flow from Saline buffer and Copper chloride solution are equal and flow from Control buffer enables complete switching between Saline buffer and Copper chloride solution into the downstream imaging area (see Figure 4D and troubleshooting Note 2).

**CRITICAL:** Avoid introducing bubbles into the device during device preparation (see also troubleshooting Note 1). Ensure that the outflow valve is open *only* when all device inlet and outlet ports are connected to tubing or blocking pins. Close the outflow valve whenever any tubing adjustments are made.

### Load animal into microfluidic device

**Timing: ∼15 min**

This step describes loading an individual animal into the microfluidic device without disturbing the fluidic set up or introducing bubbles.

13. Gently transfer one young adult *C. elegans* to a small (35 or 60 mm) unseeded Nematode Growth Medium (NGM) agar plate.
14. Flood the NGM plate about halfway to the rim with Saline buffer.
15. Fill the worm loading syringe with ∼1 mL Saline buffer and purge any bubbles.
16. Draw an animal into the worm loading syringe.
  a. With a drop-to-drop connection, submerge the tip of the worm loading syringe under the saline solution.
  b. Keeping the tip of the syringe submerged, gently draw the swimming animal into the tubing section of the worm loading syringe. Do not draw the animal into the syringe barrel.
17. Close the outlet valve to cease flow through the microfluidic device.
18. Connect the worm loading tubing to the microfluidic device.
  a. Remove the blocking pin from the worm loading inlet and allow a small droplet to form by briefly opening the buffer reservoir valve.
  b. Insert the worm loading tubing with a drop-to-drop connection.
  c. Remove excess liquid from the glass by aspiration or paper towel.
19. Establish fluid flow in the microfluidic arena.
  a. Open the outlet valve to resume flow. Typical flowrate is about 1 μL/s or ∼4 mL/h, and is proportional to the difference in fluid height between inlet and outlet reservoirs.
  b. Adjust flowrate by raising or lowering reservoirs. Behavior is generally insensitive to flowrate (Albrecht and Bargmann, 2011).
20. Insert worm into the microfluidic arena.
  a. Verify that saline buffer is present in the imaging arena (deactivate stimulus valve if not).
  b. Apply gentle pressure to the worm loading syringe plunger until the animal enters the arena.
21. Monitor the animal for 1–2 min to ensure behavior is normal and the animal is healthy (see troubleshooting Note 3).

### Set testing parameters and run experiment

**Timing: 5–10 min setup, 12 h duration**

This step describes the final steps of setting experimental parameters to determine sleep/wake state prior to running the closed-loop script.

22. Capture a single image frame of the arena under brightfield illumination with the animal visible.
23. Using the “**ImageJ**” toolbar in “**Micro-Manager,**” import the image frame at 25% scale.
24. Using the rectangle tool, draw a box spanning the outermost points of the arena where the animal will roam. This selection box will be the area of interest used for analyzing animal movement. Record the dimensions (x, y, w, h) of the rectangle, visible in the ImageJ toolbar window (Figure 5).

**Figure 5:**
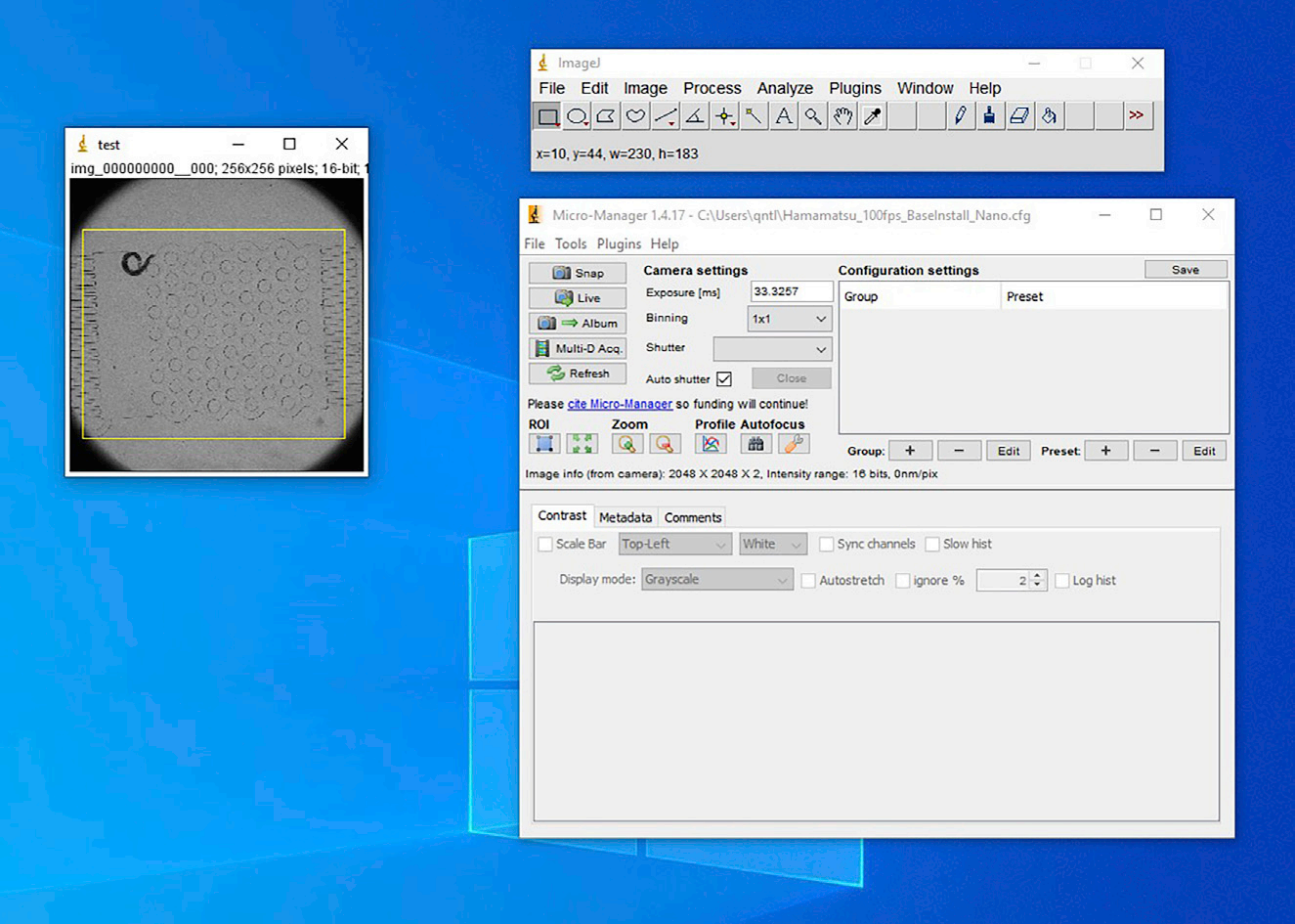
Micro-manager software and selection of the arena area-of-interest for sleep detection Using the rectangle tool within ImageJ, drag a box across the entire arena, record the dimensional parameters, x and y position (of top left corner) and width (w) and height (h), and enter them into the script code.
25. Open “**SleepDetection.ijm**” and edit the variables x, y, w, and h near the top to define the selected area of interest, then save the file.
26. Locate the “**User Defined Closed Loop Settings.txt**” file on the computer and set parameters as desired (Figure 6). Detailed information on each parameter is available within the file and in the “**ClosedLoopSleepStim.bsh**” script.

**Figure 6:**
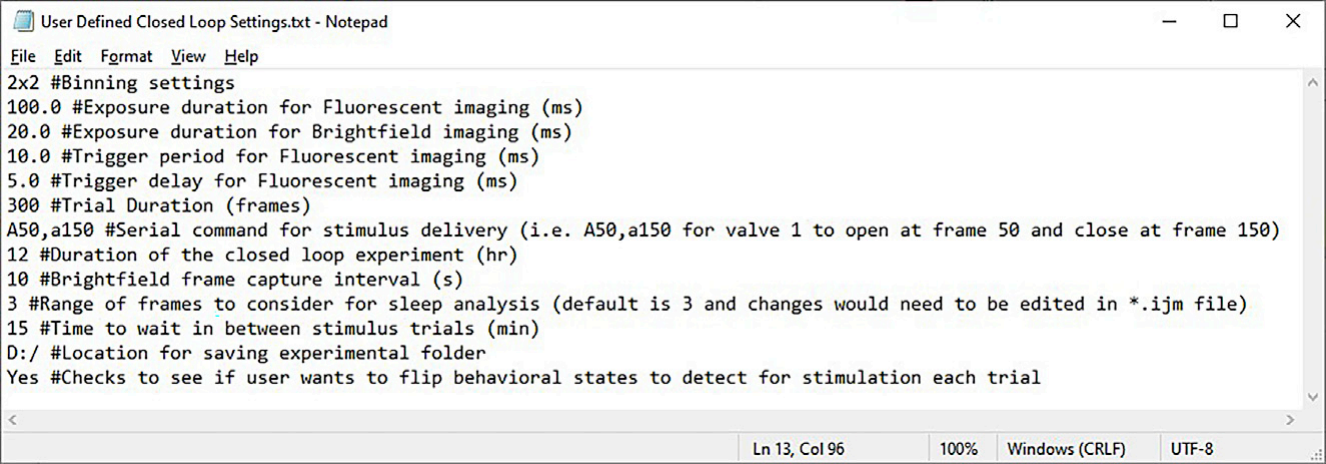
Sample experiment settings file These sample settings define imaging resolution (bin 2), framerate (100 ms exposure = 10 frames/s), and illumination timing (20 ms for brightfield, 10 ms for fluorescence with 5 ms delay from exposure onset). Stimulation recordings capture 300 frames (or 30 s at 10 fps), and the stimulation program is a 10 s chemical pulse (valve 1 opens from frame 50–150 or from 5–15 s within each recording trial). The entire experiment lasts 12 h. Sleep state changes are monitored using a 10-s interval and changes must persist for 3 intervals (i.e., 30 s) to register a transition. After each stimulation trial, 15 min should elapse before triggering a new stimulation (regardless of any transitions within this time delay), and the next trigger shall begin following the opposite transition (i.e., Sleep-to-wake follows Wake-to-sleep).
27. Run the “**ClosedLoopSleepStim.bsh**” within Micro-Manager to initiate the experiment.

***Note:*** Occasionally animals try to escape the microfluidic arena through the worm entry port (see troubleshooting Note 3). Most often this occurs at the beginning of the experiment, so it is advised to monitor progress during the first few minutes after loading the animals. Use the worm loading syringe to gently push the animal back into the field of view if the animal attempts to escape the arena, or load a new animal if necessary. Once the animal is well established in the arena, the experiment can run unattended.

## Expected outcomes

**Figure 7:**
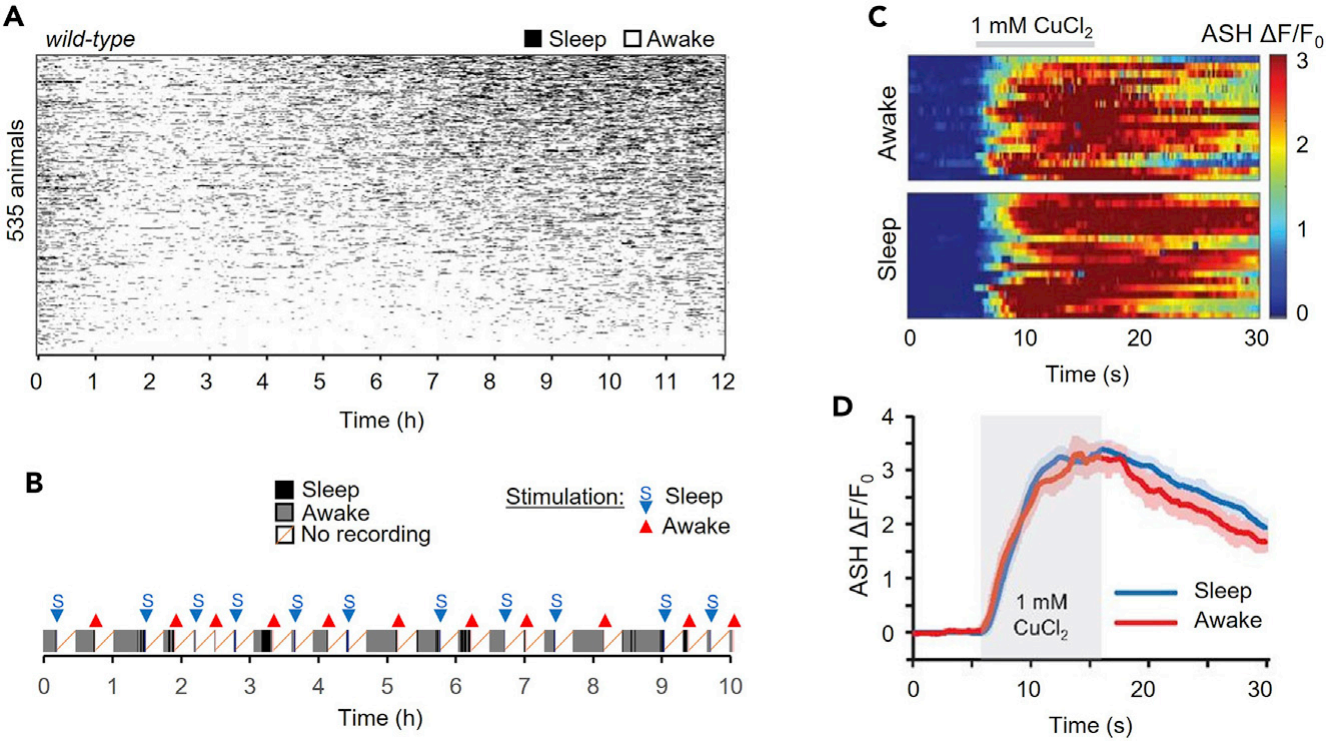
Example behavioral and neural data (A) Raster plot of sleep events (black) over 12 h, sorted by total sleep fraction (n = 535 animals, Lawler et al., 2021). Note that these data were obtained using a large population microfluidic device, not described in this protocol. (B) Example single animal behavior in a typical 10-h closed-loop experiment indicating automatically triggered neural recording trials. Sleep and awake states are indicated by black and gray shading. Recordings were triggered one minute after a sleep state change, followed by 15 min of rest (white shading). (C) Heatmap of individual ASH neural responses to 10 s stimulation with 1 mM CuCl_2_ (gray bar), in sleeping and awake animals. (D) Average ASH neural responses in sleep and awake states from panel C (n = 18 sleep, 17 awake). Shading represents s.e.m. No change is observed in ASH sensory responses between the sleep and wake states, indicating a consistent sensory response. Other neurons may change in peak magnitude, duration, rise time, decay rate, or other metric across sleep states.

Adult *C. elegans* sleep behavior varies greatly even across a population of genetically identical wild-type animals under no stimulation (Figure 7A). On average, about 10%–30% of unstimulated wild-type adult *C. elegans* animals should be in a sleep state at any moment, and median sleep bout duration is about 1.5–2 min (Lawler et al., 2021). With a moderate stimulus (such as 1 mM CuCl_2_), a typical 12 h experiment should yield ∼12–24 sleep/wake transitions or approximately one transition every 30–60 min (Figure 7B). If sleep/wake event yield is too low with a stronger stimulus, sleep events can be induced via deoxygenation (such as using sodium sulfite, see Lawler et al., 2021). Many factors can influence sleep dynamics, including environmental conditions (see troubleshooting Note 4).

The closed-loop script organizes behavioral videos and neural stimulation sets into a designated save location. All behavioral videos are contained in “TimeLapse” folders separated between stimulations. All stimulus captures are labeled in “Stimulus” folders with the detected behavioral state indicated. Sleep state predictions were found to be 93.4% accurate compared to human observation (Lawler et al., 2021). It is recommended to verify correct identification of behavioral transitions prior to sleep state analysis. For example, view behavioral videos and verify that sleep bout videos show minimal motion and sleep-specific posture (Lawler et al., 2021) and awake videos do not include sleep periods (see troubleshooting Note 5).

## Quantification and statistical analysis

To quantify neural fluorescence, **NeuroTracker** (Larsch et al., 2013) ImageJ software tracks the position of the neuron over time and integrates fluorescence intensity of the soma within a small integration box (available with usage instructions at https://github.com/albrechtLab/Neurotracker). Use active behavior tracking settings as animals frequently move during the stimulation period. Choose an integration box size that just encloses the neuron soma, typically 4 × 4 pixels or 6 × 6 pixels.

NeuroTracker outputs a text file containing neural tracking data over time for each animal, including centroid position, integrated fluorescence intensity, and background level. A MATLAB script “**NeuroTrackerSummary.m**” can be used to read, organize, and visualize neural fluorescence data across individuals (Figure 7C) or averaged across groups such as sleep and wake states (Figure 7D). Neural fluorescence (F) is calculated by subtracting the background (median intensity of an annulus surrounding the neuron) from average fluorescence intensity in the integration box. To compare across animals with different GCaMP expression, intensity measurements are normalized to a baseline consisting of the first 4 s of each trial before stimulation (F_0_). The normalized activity over baseline, ΔF/F_0_ = (F – F_0_)/F_0_, can be averaged across individuals and population groups. For statistical comparisons, it is recommended to reduce data per trial to metrics such as peak calcium activity, area-under-curve (AUC), baseline F_0_, rise time, and decay time. These values can then be compared statistically across sleep states, perturbations, or other experimental groups.

## Limitations

Unlike lethargus states between well-timed larval stages, adult sleep events are not time synchronized and occur randomly. Since chemical stimulation is applied uniformly across the entire arena in the neural imaging device, the methods described above are limited to one animal at a time. Additionally, sleep state is determined by a whole-field image subtraction, necessitating one animal per image field. Due to this reduced throughput, it is advised to first determine the overall sleep response to new genetic or chemical perturbations using a high-throughput microfluidic population behavior assay (Lawler et al., 2021), then analyze neural responses as described here using identical conditions. Alternatively, timed stimulus pulses can be delivered and resulting neural responses can be grouped post hoc based on each animal’s sleep state. However, while this method allows for analysis of many animals at once, it does not control for timing between state transitions and stimulation tests.

To monitor free-roaming behavior without camera tracking, a low-magnification (5×) objective is used such that the entire 3 mm × 3 mm microfluidic arena is contained within the field of view. Calcium imaging at this magnification is sufficient for tracking single neural activity, or a few neurons spaced at least 10–20 μm apart. This resolution is not sufficient for whole-brain imaging. Additionally, some neural imaging *C. elegans* strains may not show sufficient expression to be clearly visible at this magnification or may have low baseline activity. For example, newer GCaMP variants (6+) increase dynamic range by decreasing baseline fluorescence, making tracking of inactive neurons more difficult without additional markers, such as a stable red fluorescent protein expressed in the same neuron.

The sleep detection script relies on detection of movement across frames, and therefore may not be suitable for detecting sleep events in mutants that have overall movement deficiencies. Adjusting movement sensitivity parameters may be sufficient for accurate state detection (see troubleshooting Note 5). Alternatively, the image processing script can be altered to assess any type of optical reporter that does not rely on movement. For example, states and trigger events could be defined by fluorescent reporters expressed in neurons, muscles, or other cells. In this way, the closed-loop sleep detection and stimulation system could be broadly adapted to automatically assess neural responses following any detectable biological phenomenon, such as a locomotory behavior, neural response, mating event, or a change in level or localization of a signaling molecule.

## Troubleshooting

### Problem 1

Air bubbles enter or form in the microfluidic chamber (steps 5 and 11).

### Potential solution

A common problem with microfluidic devices is the presence of air bubbles within the liquid channels, which affect fluid flow, animal behavior, and microscope imaging. Bubbles can either be present at the beginning of an experiment or form during the experiment. To avoid initial bubbles, the microfluidic devices should be degassed in a vacuum chamber before filling such that any bubbles present upon fluid inflow are absorbed into the gas-permeable PDMS material. Extended vacuum duration, and quickly loading fluid upon release from vacuum, will accelerate this process. Next, it is important to avoid flow of air bubbles from the tubing and Luer fittings. Thoroughly purge air from reservoirs and inlet tubing, using vigorous flicks of the Luer fittings to dislodge any bubbles, and make careful drop-to-drop connections at the inlets as described in Lagoy and Albrecht (2015) and Reilly et al. (2017). Finally, to avoid bubble growth during a long experiment, ensure that the chamber is kept at a net positive pressure relative to atmosphere by keeping the inlet and outlet reservoir liquid levels above the device. While it is possible to remove bubbles by pressurizing the chamber, it is usually faster to open, clean, reassemble, and refill the microfluidic device.

### Problem 2

Fluids do not switch properly during stimulation (step 12).

### Potential solution

Proper fluid switching ensures that the stimulus is delivered reliably to the animal regardless of its position within the arena. During the setup procedure, it is recommended to include a temporary stimulus solution containing fluorescein or other visible dye, and ensure that upon valve actuation the entire arena fills with stimulus solution (see Figure 4D). Imbalanced flow is indicated by a buffer stream at the top or bottom of the arena during valve actuation, or a stimulus stream without valve actuation. To balance flows, ensure that the tubing lengths and diameters are identical, and that reservoirs are positioned such that fluid heights are level. Slight adjustments in flow balance can be made by raising or lowering reservoirs. Note that any fluorescent dye in the stimulus or buffer solutions should be removed after flow balancing, as it can affect tracking of neural activity. One convenient method is to draw a small amount of dye (∼20–50 μL of 0.1 μg/mL fluorescein) into the tubing of either the stimulus or buffer reservoirs using the priming syringe. Flow balance can then be monitored and adjusted until the dye fluid has passed. Fluorescein added to the control fluid should never pass through the arena. Ensure that the control fluid only exits via the bypass channels around the arena, with equal stream width in the upper and lower channels as the valve is actuated.

### Problem 3

Animal escaped the microfluidic arena (step 21).

### Potential solution

Animals may try to escape the arena in search of food or other attractants. Physical barriers upstream and downstream keep the animal actively roaming in the field of view. Animals that escape through these barriers may be physically smaller than a young adult stage wild-type *C. elegans*. For example, mutants may grow more slowly and require more time to reach the desired size. Additionally, animals may escape if the device is not sealed properly or is damaged. Carefully check the device for damage, especially at the barriers using a stereomicroscope, and ensure all surfaces of the microfluidic device and glass pieces are fully cleaned of debris prior to assembly. Minimize syringe force when loading the animal, as overpressure may disrupt the seal between the device and the glass slide.

An animal may also escape the microfluidic arena through the worm loading port located upstream in the device, especially if fluid flow is slow or stopped. Ensure that sufficient downstream flow is present to keep animals within the micropost array of the arena. A typical flowrate of 1 μL/s is sufficient, corresponding to about 50 cm height between inlet and outlet reservoir levels. Monitor the beginning of each experiment, and if an animal enters or nears the worm loading port, gently apply pressure to the worm loading syringe to push the animal back into the micropost array.

### Problem 4

The animal does not sleep (expected outcomes section).

### Potential solution

Sleep in adult *C. elegans* is sensitive to many environmental factors and varies substantially across individuals. A lack of sleep may result from unintended stimulation to the animal. Stimulation from light (especially blue and ultraviolet light), chemical stimuli, and mechanical perturbations can limit sleep. Ensure that the animal is not exposed to any unnecessary blue/UV light, that buffers are freshly prepared, that animals are loaded into arenas filled with buffer (not stimulus), that flow through the microfluidic device is gentle, and that animals exhibit natural crawling movement. If animals do not sleep after the first sleep transition stimulation, the stimulus concentration may be too high. Additionally, rough handling of the animal prior to loading can also lead to a diminished sleep response.

### Problem 5

The script does not correctly identify sleep states (expected outcomes section).

### Potential solution

Sleep detection follows an image processing algorithm that may require adjustment based on image quality and lighting to correctly identify the animal. To determine adjustments, observe the result of each step in the segmentation process “**SleepDetection.ijm**” using the ImageJ software. For example, segmentation of the animal from the microfluidic environment requires thresholding, by default using Otsu’s method to isolate the foreground (animal) from the background of the image. Depending on lighting conditions, other automatic or manual thresholding methods may be more effective. After thresholding, the script identifies a valid animal assuming a consistent pixel resolution to size the animal. The script by default expects an image resolution of ∼350 pixels/mm when at full scale (∼90 pixels/mm at 25% scale), and this parameter should be adjusted for other image resolutions or animal sizes.

## Resource availability

### Lead contact

Further information and requests for resources and reagents should be directed to Dirk R. Albrecht (dalbrecht@wpi.edu).

### Materials availability

*C. elegans* strains are available from the *Caenorhabditis* Genomics Center, or from the corresponding author of published works. Microfluidic design files are available upon request to the lead contact.

## Data Availability

Custom control scripts and analysis software are available at Github (https://github.com/albrechtLab/ClosedLoopStimulation and https://github.com/albrechtLab/Neurotracker).
